# Side-Effects on the Renal function of Cytoreductive Surgery with Hyperthermic Intraperitoneal Chemotherapy

**DOI:** 10.7150/ijms.97774

**Published:** 2024-09-30

**Authors:** Diego Beltrán, María-Consuelo Pintado, Ana Oñoro, Inmaculada Lasa Unzúe, Remedios Gómez Sanz, Manuel Diez Alonso, Miguel A. Ortega, Melchor Álvarez de Mon, Emilio Nevado Losada, Alberto Gutierrez Calvo

**Affiliations:** 1 Intensive Care Unit. Hospital Universitario Príncipe de Asturias. 28805 Alcalá de Henares, Madrid, Spain.; 2 Department of General and Digestive Surgery. General and Digestive Surgery. Hospital Universitario Príncipe de Asturias. 28805 Alcalá de Henares, Madrid, Spain.; 3 Department of Medicine and Medical Specialities (CIBEREHD), Faculty of Medicine and Health Sciences, University of Alcalá. 28801 Alcalá de Henares, Spain.; 4 Ramón y Cajal Institute of Sanitary Research (IRYCIS), 28034 Madrid, Spain.; 5 Inmune System Diseases-Rheumatology and Internal medicine Service. Hospital Universitario Príncipe de Asturias (CIBEREHD), 28806, Alcalá de Henares, Spain.

**Keywords:** cytoreductive surgery, hyperthermic intraperitoneal chemotherapy, acute kidney in-jury, morbidity, mortality

## Abstract

**Background:** Acute kidney injury (AKI) is a frequent complication in patients undergo-ing cytoreductive surgery (CRS) with hyperthermic intraperitoneal chemotherapy (HIPEC) due to a combination of several factors: increased intra-abdominal pressure, heat stress and drug tox-icity.

**Methods:** Patients admitted to Intensive Care Unit after CRS and HIPEC during 129 months. Data recorded were: demographic characteristics; severity of illness, haematology and basic chemistry panels (renal function and electrolytes), type of cancer and extension, HIPEC drug and temperature, fluid balance, ICU and hospital stay and mortality.

**Results:** 123 patients were included, only 4.9% developed AKI. AKI was more frequent on patients with ovarian cancer or in those who received doxorubicina as intraperitoneal chemotherapy; also, among those who had higher positive fluid balance during surgery, had higher SOFA or were under mechanical ventilation at ICU admission. There were not differences in mortality according to the development of AKI. Electrolyte disorders appeared in 95.8% of the patients, mainly hypocalcemia and hypokalemia.

**Conclusion:** In our study, the incidence of AKI has been low. The presence of hydroelectrolytic alterations and polyuria was very frequent. The type of cancer, no mitomycin-based regimens and positive fluid balance during surgery were factors that suggest increased risk of AKI. However, although patients with AKI were clinically worse it was not associated with higher mortality.

## 1. Introduction

Cytoreductive surgery (CRS) combined with hyperthermic intraperitoneal chemotherapy (HIPEC) is a therapeutic approach involving removal of visible tumor, followed by the infusion of heated chemotherapy into peritoneal cavity. This approach aims to achieve very high local concentration of chemotherapy in order to optimize tissue penetration and effectiveness 1, 2. However, this procedure may be extensive and prolonged, often involving significant fluid shifts and blood loss due to tissue resection and chemotherapy solutions. Additionally, it induces hemodynamic and metabolic alterations caused by heat exposure, as well as, systemic toxicity due to absorption of chemotherapy agents or their carrier solution^3^.

Several side-effects and complications of this treatment have been reported, involving the digestive, hematopoietic, circulatory, metabolic and urinary systems. These effects exhibit inter-individual variability and are influenced by factors such as the specific agents and the dose used 4. The presence of these complications is associated with a poorer prognosis^5^.

Acute kidney injury (AKI) is a frequent complication in patients undergoing HIPEC, especially when cisplatin is used as a chemotherapeutic agent^2^. The incidence of AKI varies between 0.8 and 48% depending on the studies^4^, ^5^. While abdominal hyperthermia itself does not seem to cause or worsen AKI, a combination of factors including increased intra-abdominal pressure, heat stress and drug toxicity contributes to its pathogenesis^6^. These factors may lead to altered water-electrolyte balance, such as hyponatremia, hypokalemia, hyperglycemia or metabolic acidosis, requiring aggressive fluid resuscitation^6^.

The primary objective of this study is to elucidate the risk factors and incidence of acute kidney injury (AKI) during the immediate postoperative period in patients undergoing cytoreduction surgery and HIPEC who were admitted to the ICU. The secondary objective is to characterize the electrolyte imbalances that may appear in these patients. This investigation aims to identify modifiable factors to develop strategies to reduce the morbidity related to HIPEC.

## 2. Materials and Methods

Observational, prospective Spanish study that included patients admitted to the ICU after undergoing cytoreductive surgery and hyperthermic intraperitoneal chemotherapy with closed technique. The study period ranged from January 1, 2013, to September 30, 2023, (129 months), with exclusion criteria applied to those who did not provide consent to participate in the study.

The following data were collected: demographic characteristics, comorbidities and Charlson index^7^. Time between the last administration of intravenous (IV) contrast and surgery, disease severity measured by APACHE II^8^ and SOFA^9^ was also documented. Laboratory tests covering haematology and basic chemistry panels, including assessments of renal function and electrolytes that were conducted upon ICU admission and subsequently on a daily basis until the day of ICU discharge. Additionally recorded were the type of cancer, the peritoneal carcinomatosis index (PCI)^10^, the drug used and the HIPEC temperature, fluid balance during surgery and ICU stay. The necessity for vasoactive support and/or mechanical ventilation, the requirement for blood products, surgical time, hospital or ICU stay and mortality were also described. In cases, where more than one value was available for a given day, the most abnormal value was recorded.

Intraperitoneal chemotherapy regimens used included mitomycin C-based regimens for carcinomatosis of appendicular, colorectal, and gastric origin, and cisplatin-based regimens for carcinomatosis of ovarian, peritoneal sarcomatosis, and mesothelioma, or combinations with other drugs. At our institution, patients are not eligible for HIPEC with cisplatin if they have an estimated glomerular filtration rate (eGFR) <60 ml/min/1.73 m^2^ or a baseline creatinine (Cr) ≥1.5 mg/dl, have end-stage renal disease, or are receiving renal replacement therapy.

We defined acute kidney injury (AKI) according to the Kidney Disease Improving Global Outcomes (KDIGO) criteria^11^ as an increase in serum creatinine (Cr) of ≥ 0.3 mg/dl within 48 h after surgery compared to baseline serum creatinine (defined as serum creatinine at hospital admission 24h before surgery) and/or an increase in creatinine compared to 48 hours earlier.

Electrolyte disorders were defined as values outside the normal reference range of our laboratory. The normal reference values for electrolytes in our laboratory were as follows: sodium 135-145 mmol/l, potassium 3.5-5.5 mmol/l, magnesium 1.5-2.3 mg/dl, phosphate 2.4-5.1 mg/dl and ionic calcium 1.15-1.35 mmol/l. Polyuria was defined as urine production greater than 3 L/day.

Management of patients during pre and intraoperative period and ICU stay was done according to standard protocols, including fluids, blood transfusions, vasoactive drugs, diuretics and glucose control.

For statistical analysis, quantitative variables with a normal distribution were compared using Student's T test and expressed as means; those with a non-normal distribution were compared using the Mann-Whitney test and expressed as medians and interquartile ranges. For qualitative variables, the chi-square test was employed, and the results were presented as number and percentages.

The Institutional Ethics and Clinical Trials Committee of the Príncipe de Asturias University Hospital approved the study on 1^st^ July 2014 (internal code OE 30/2014).

No financial or logistical support was received from any institution other than the service or hospital where the study was carried out, to carry out the study. There were also no fees or material incentives for participation in the study for either the patients or the researchers who took part in the study. The study (FIS-PI21/01244) was supported by the Instituto de Salud Carlos III (grant no. Estatal de I + D + I 2020-2027) and co-financed by the European Development Regional Fund “A way to achieve Europe”, as well as P2022/BMD-7321 (Comunidad de Madrid) and ProACapital, Halekulani S.L. and MJR, solely and exclusively for the payment of publication fees.

## 3. Results

124 patients were admitted to our ICU after CRS and HIPEC during the study period. 123 were included, as one patient opted not to participate.

The mean age was 59.50 ± 10.52 years, almost half being male (49.6%). 104 patients (84.6%) had cancer of digestive origin, being the rest of ovarian origin. The median peritoneal carcinomatosis index for all study patients was 7.00 (3.00-14.00) (Table 1).

The last dose of IV contrast for all was received, at least, 1 month before surgery. 65 patients (52.8%) received preoperative chemotherapy; all of them received the last dose at least 2 months before surgery.

Median duration of surgery was 9.52 ± 2.00 hours, during which patients maintained a mean positive fluid balance of 4314 ± 1562 ml and 17 patients (13.8%) required blood transfusion.

At ICU admission, the median APACHE II and SOFA score were 8.50 (6.000-12.00) and 1.00 (1.00-2.00) respectively. 6 patients (4.9%) were under mechanical ventilation, and 9 (7.3%) needed vasoactive support during or at ICU admission (Table 1).

During ICU admission only 6 patients (4.9%) developed acute kidney injury; all of them in stage 1 according to KDIGO classification^11^. AKI was more frequent in patients with ovarian cancer or those who received doxorubicin as intra-peritoneal chemotherapy. At ICU admission, patients who developed AKI had a higher positive fluid balance during surgery, a higher SOFA score at ICU admission and were on mechanical ventilation (Table 1).

A total of 95.8% of the patients included in the study developed some kind of electrolyte disorder, with most of them being asymptomatic. Only one patient developed ventricular tachycardia due to hypomagnesemia. There were no differences in the incidence of electrolyte disorders between those who developed acute kidney injury and those who did not, except in the case of hypokalemia. Contrary to what might be expected, hypokalemia occurred more frequently in patients who developed acute kidney injury (Table 2).

**Figure 1:**
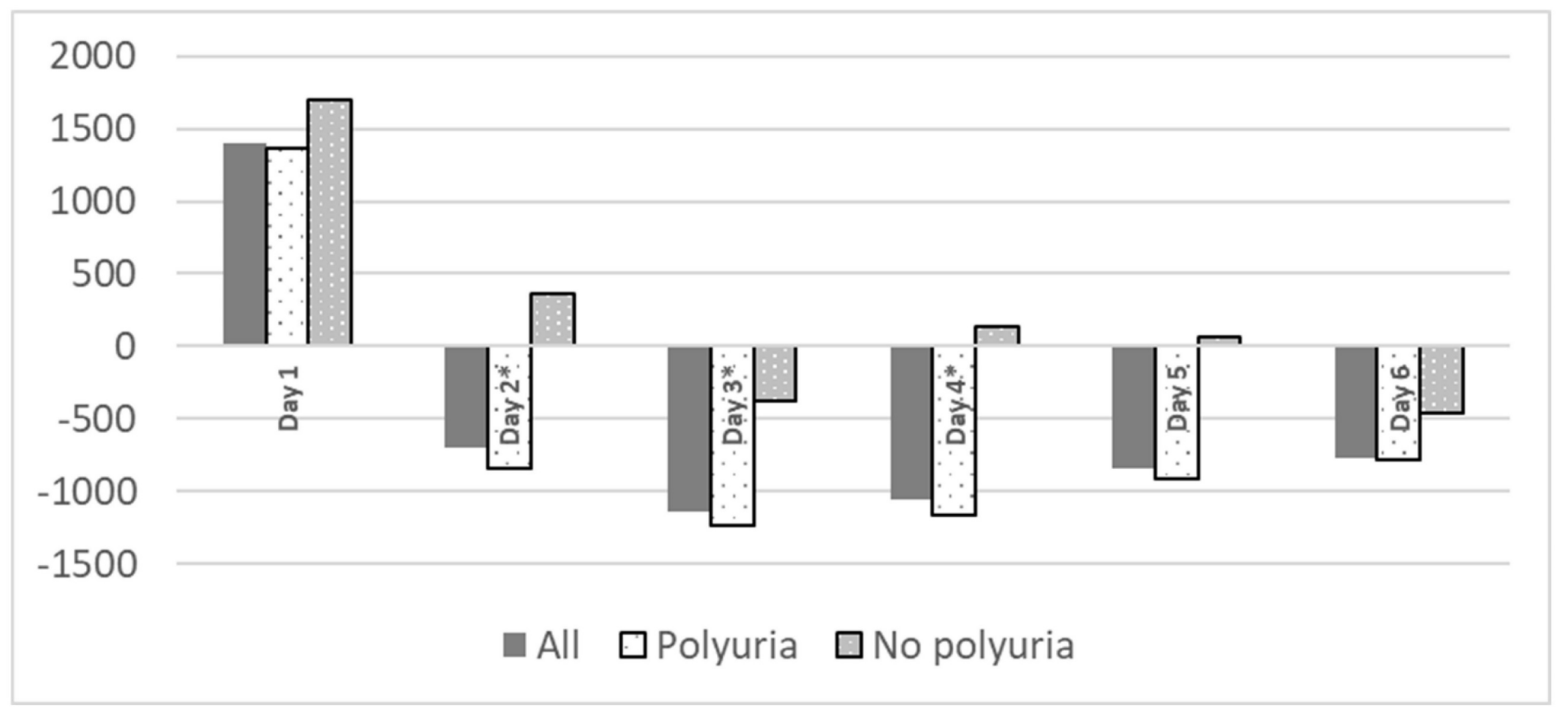
Fluid balance during ICU stay related to polyuria. Fluid balance expressed as ml/ day. *p< 0.05.

Polyuria is common in postoperative period, mainly from third day, with no significant differences related to the development of renal failure (table 3). Polyuria was not associated to fluid balance during surgery (4262 ± 1529 ml on patients who developed polyuria vs. 4723 ± 1864ml, p= 0.425). No patient received diuretics during their ICU stay. Patients with polyuria exhibited a statistically significant more negative water balance (Figure 1).

66 (53.7%) patients developed respiratory complications, not related to acute kidney injury (Table 3).

During ICU admission 10 patients (8.1%) manifested infectious complications, including pneumonia (2 cases), urinary tract infection (1 case), tracheobronchitis (1 case), Clostridium difficile diarrhea (1 case) and fever with negative cultures (5 cases). There were no significant differences observed in relation to the development or not of acute kidney injury.

The median ICU stay was 5 (4.0-5.0) days, and the median hospitalization was 10.0 (8.0-13.0) days. One patient (0.8%) died during ICU stay due to massive pulmonary embolism, none during hospital stay after ICU admission. There were no differences in ICU or hospital stay based on development or not of acute kidney injury (Table 4).

## 4. Discussion

The incidence of AKI in our study was 4.9%, being much lower than others reported in the literature, where it is approximately 20%^6^, ^12^ with a significant range of variability up to 48%^13^. This wide difference between studies can be attributed to various factors, such as the type of tumor, the chemotherapeutic regimen employed (cisplatin-based regimens have been associated with a higher risk^6^, ^12^), and variations in the definition of acute renal failure. For instance, Carias *et al.*^14^ who used the same AKI definition, reported an incidence almost three times higher (12.6%) in the Portuguese population, despite using similar HIPEC regimens and indications as ours.

Renal injuries associated with HIPEC have multiple contributing factors and it remains controversial whether they are caused by toxicity of chemotherapeutic agents or changes in the patient's renal perfusion^4^. When analyzing the reasons behind the low incidence in our sample, one possibility is the low rate of preoperative chronic renal failure (0.8%) which has been identified as a risk factor for AKI in several previous studies^14^, ^15^. Unlike findings from other studies, we observed that other comorbidities, such as diabetes^16^ and hypertension^15^, which are known to play a role in AKI associated with HIPEC, did not show significant differences, with a similar Charlson index^7^ in both study groups.

We did not find that other factors occurring during CRS and HIPEC surgery, which have also been related to the development of AKI after surgery, such as the PCI (Peritoneal Carcinomatosis Index)^10^,^6^, the duration of surgery^6^, ^17^ and blood product transfusion^6^, ^18^. Despite their potential implications for a more complex surgical course, our findings did not show significant relationships with the occurrence of AKI in our sample.

Regarding the chemotherapeutics agents used, it is well known that the use of cisplatin is associated to significant renal toxicity^6^, ^12^. Cisplatin damages the proximal renal tubules and influences renal regulatory function^19^. In fact, the existing literature indicates that acute kidney injury (AKI) is a common occurrence in HIPEC regimens that include cisplatin^6^, ^12^. Liesenfeld *et al.*^13^ described that treatment with cisplatin-containing HIPEC regimens represents a significant risk factor for HIPEC-related AKI (p<0.001). In another study performed by Hakeam *et al.*^20^, they specifically documented the occurrence of cisplatin-induced nephrotoxicity after HIPEC and determined that 3.7% of their patients experienced AKI according to the consensus RIFLE criteria. In our sample, 43.1% of the total patients received cisplatin. However, we did not find a statistically significant relationship between cisplatin and AKI. This could be due to the low incidence of AKI and the near absence of previous chronic kidney disease which could have diminished the impact of these factors. We found that AKI was more frequent in patients who received doxorubicin as intra-peritoneal chemotherapy, maybe due to all of them received doxorubicin together with cisplatin. In the univariate analysis the digestive origin of carcinomatosis in HIPEC was statistically significantly related to low incidence of AKI. This could be linked to the regular use of mitomycin C regimens in this type of cancer, and a lower use of cisplatin.

On the other hand, in our sample, AKI was statistically significantly associated to a higher positive fluid balance during surgery. Rather than being a direct cause of AKI, the need for further expansion with volume during surgery may reflect the presence of a worse hemodynamic situation in the context of greater fluid loss, bleeding, oliguria or increased capillary permeability during surgery (unfortunately, intraoperative data were not collected). This could explain their admission to the ICU with a statistical tendency to have worse SOFA^9^ and worse APACHE II^8^ compared to patients who did not develop AKI, as well as more frequent admission to invasive mechanical ventilation. Although the type of intraoperative fluid strategies was not specified, it should be noted that the hospital anesthetic protocol for this type of surgery is a strategy guided by intraoperative fluid objectives through invasive hemodynamic monitoring systems (Vigileo®/FloTrac®). Recommendations to prevent acute renal injury induced by cisplatin include the use of furosemide and mannitol to favour elimination of the drug in the urine^21^, ^22^. No diuretics were administered in the ICU. Although a positive water balance was required first day of admission, from the second day onward, it was balanced or negative, conditioned by the presence of polyuria in some patients. This differs from the studies by Liesenfeld *et al.*^13^, ^23^ where no significant differences in water balance were observed in the first postoperative days between those with AKI and those without AKI.

A frequent cause of death related to CRS and HIPEC treatment is sepsis and infection^24^. Our rate of infectious complications is low (8.1%) compared to other studies reporting rates of about 45%^24^, ^25^, with no significant differences between AKI and non-AKI groups. On the other hand, the rate of respiratory complications was 53.7% (including acute respiratory failure, pleural effusion, atelectasis or others) being comparable to other studies^26^, ^27^ and without differences between AKI and non-AKI patients.

It has been theorized that hydro-electrolytic alterations associated with HIPEC occur due to the high temperatures used during the procedure. These temperatures can cause damage not only to malignant cells which are the target of the therapy, but also to healthy tissues, leading to disruptions in its proper functioning, specifically in the ionic exchange. In addition, chemotherapeutic solutions with low permeability may result in ionic losses to the exterior^28^-^30^ through osmotic mechanisms. In our sample, ionic alterations were observed in 95.8% of the cases, with most frequent being hypocalcemia (86.2%) and, to a lesser extent, hypokalemia (40.7%) and hypomagnesemia (25.2%). These alterations align with findings in other studies^14^, ^20^. It is plausible that there is a correlation between these ionic alterations and the use of chemotherapeutic agents^29^, ^31^ (such as hypomagnesemia and hypokalemia due to cisplatin^32^, ^33^), thermal changes, the significant need for intraoperative fluid therapy (dilutional hypomagnesemia^20^ or metabolic acidosis induced by normal saline), the requirement for blood products (transfusion-induced hypocalcemia) and, in our case, the presence of a significant inappropriate polyuria generated from the second postoperative day onwards, as shown in Figure 1. This polyuria, observed in both groups and not attributed to diuretics, hyperglycemia, or recovery from renal failure, presented a challenge in achieving a balanced water balance in the entire patient cohort. We lack data on its rate of appearance in the immediate postoperative period from previous studies.

Finally, the most surprising aspect in our sample was not only the low incidence of AKI but also the low mortality in the ICU and in the hospital. Both groups had a similar length of stay in the ICU of at least 5 days (according to institutional protocol) and 10 days in hospital. Over the 10 years of the study, only one death (<1%) occurred, which is consistent with findings in other studies^25^, ^34^. These outcomes shows the adequate selection of patients and treatment, the safety of the procedure, the good surgical techniques, the effective perioperative management and the postoperative monitoring in the ICU in patients who require CRS and HIPEC.

The present study has several limitations, including its single-center nature, the absence of pre and intraoperative data on factors that can influence the development of AKI such as time to HIPEC infusions for various drugs, use of renal protective agents, extent of peritoneal resections, intraabdominal pressure during surgery, severity of surgery, number and extend of hypotensive episodes, drugs used during pre an intraoperative period, usual previous medications, the existence of various combinations of regimens for HIPECs and the inclusion of different types of oncologic patients. Given the low number of patients with AKI, it was not possible to perform a multivariate analysis.

However, it also has the strengths, such as a substantial number of patients and the implementation of strict clinical and analytical follow-up with a minimum of 5 days in the ICU according to hospital protocol. To our knowledge, this is one of the first studies to analyze AKI and ionic alterations in immediate postoperative period after CRS and HIPEC in Spain. There are other studies realized in Spain focusing on the effect of imipenem/cilastatin in the prevention of AKI in patients with advanced cancer undergoing HIPEC with cisplatin, they found a higher incidence of some degree of AKI (defined according RIFLE classification) than in our study: 25.5% in control group and 22.8% in interventional group^35^.

In conclusion, the incidence of AKI in our patients with Hyperthermic Intraperitoneal Chemotherapy (HIPEC) over these 10 years has been low. The type of cancer, no mitomycin-based regimens and the higher need for fluids in the operating room was associated with a higher risk of AKI in the immediate postoperative period, possibly reflecting the presence of hypotension, bleeding and/or oliguria during intraoperative period. However, despite the fact that patients with AKI were clinically worse (higher SOFA^9^, worse APACHE II^8^ and greater need for mechanical ventilation) it was not associated with higher mortality. Both groups experienced a high prevalence of dyselectrolytemias, with hypokalaemia and hypocalcemia being the most frequent, but without evident clinical impact. Further studies are needed to determine the role of polyuria after HIPEC and its association with electrolyte disturbances. Given our low mortality and incidence of AKI, we recommend that all patients undergoing Cytoreductive Surgery (CRS) and HIPEC should be monitored in the immediate postoperative period in the Intensive Care Unit (ICU) as a strategy to reduce the occurrence of AKI, and as part of adequate perioperative management, along with an adequate patient and treatment selection and surgical technique.

## Figures and Tables

**Table 1:** Patient characteristics.

	All (n = 123)	AKI (n = 6)	Non-AKI (n = 117)	p
Age, years (mean +/- S.D.)	59.50 ± 10.52	62.00 ± 8.39	59.38 ± 10.63	0.553
Male sex (number and percentage)	61 (49.6%)	2 (33.3%)	59 (50.4%)	0.680
Type of cancer (number and percentage)				0.047
Digestive cancer	104 (84.6%)	3 (2.9%)	101 (97.1%)	
Ovarian cancer	19 (15.4%)	3 (15.8%)	16 (84.2%)	
Baseline serum creatinine, mg/dl (mean and S.D.)	0.77 ± 0.19	0.72 ± 0.22	0.77 ± 0.19	0.462
Charlson score (median and I.R.)	6 (6-6)	6 (6-7)	6 (6-6)	0.206
Comorbidity				
Diabetes mellitus	17 (13.8%)	1 (16.7%)	16 (13.7%)	1.000
Hypertension	52 (42.3%)	3 (50.0%)	49 (41.9%)	0.697
Dyslipemia	47 (38.2%)	1 (16.7%)	46 (39.3%)	0.405
Obesity	9 (7.3%)	1 (16.7%)	8 (6.8%)	0.372
Previous respiratory disease	10 (8.1%)	0 (0.0%)	10 (8.5%)	1.000
Previous cardiological disease	4 (3.2%)	0 (0.0%)	4 (3.5%)	1.000
Previous kidney disease	1 (0.8%)	0 (0.0%)	1 (0.9%)	1.000
Malnutrition	18 (14.6%)	1 (16.7%)	17 (14.5%)	1.000
Peritoneal Carcinomatosis Index (median and I.R.)	7.00 (3.00-14.00)	7.00 (3.25-17.50)	7.00 (3.00-14.00)	0.960
Intraperitoneal chemotherapy (number and percentage)*				
Mitomycin C	87 (70.7%)	1 (16.7%)	86 (73.5%)	0.008
Cisplatin	53 (43.1%)	3 (50.0%)	50 (42.7%)	1.000
Doxoribucin	19 (15.4%)	3 (50.0%)	16 (13.7%)	0.047
Oxaliplatin	16 (13.0%)	2 (33.3%)	14 (12.0%)	0.174
Fluorouracil	9 (7.3%)	1 (16.7%)	8 (6.8%)	0.372
Duration of surgery, hours (median and I.R.)	9.52 ± 2.00	10.80 ± 2.05	9.45 ± 1.99	0.143
Temperature, degrees centigrade (median and I.R.)	43 (42-43)	43 (42-43)	43 (42-43)	0.983
Fluid balance during surgery, ml (mean +/- S.D.)	4314 ± 1562	6206 ± 2629	4201 ± 1430	0.012
Transfusion of blood products during surgery (number and percentage)	17 (13.8%)	2 (33.3%)	15 (12.8%)	0.193
APACHE II (median and I.R.)	8.50 (6.00-12.00)	14.50 (7.50-18.25)	8.00 (6.00-12.00)	0.061
SOFA at ICU admission (median and I.R.)	1.00 (1.00-2.00)	3.50 (1.00-6.25)	1.00 (0.50-2.00)	0.027
Mechanical ventilation at ICU admission	6 (4.9%)	2 (33.3%)	4 (3.4%)	0.027
Vasoactive support at ICU admission	9 (7.3%)	1 (16.7%)	8 (6.8%)	0.372

Abbreviations: AKI: acute kidney injury. S.D.: standard deviation. IR: interquartile range. ICU: in-tensive care unit. *Patients received more than 1 chemotherapy drug.

**Table 2:** Electrolyte disorders.

	All (n = 123)	AKI (n = 6)	Non-AKI (n = 117)	p
Electrolyte disorders	115 (95.8%)	6 (100.0%)	109 (95.6%)	1.000
Hyponatremia	27 (22.1%)	2 (33.3%)	25 (21.4%)	0.613
Hypernatremia	4 (3.3%)	1 (16.7%)	3 (2.6%)	0.183
Hypokalemia	50 (40.7%)	5 (83.3%)	45 (38.5%)	0.040
Hyperkalemia	3 (2.4%)	1 (16.7%)	2 (1.7%)	0.140
Hypomagnesemia	31 (25.2%)	2 (33.3%)	29 (24.8%)	0.641
Hypermagnesemia	10 (8.1%)	1 (16.7%)	9 (7.7%)	0.405
Hypophosphatemia	29 (23.6%)	1 (16.7%)	28 (23.9%)	1.000
Hypocalcemia	106 (86.2%)	6 (100.0%)	100 (85.5%)	0.595
Hypercalcemia	1 (0.8%)	0 (0.0%)	1 (0.9%)	1.000

All results are expressed as number and percentage. AKI: acute kidney injury.

**Table 3:** Respiratory complications according to acute kidney injury.

	All (n = 123)	AKI (n = 6)	Non-AKI (n = 117)	p
Acute respiratory insufficiency	55 (44.7%)	3 (50.0%)	52 (44.4%)	1.000
Pleural effusion	24 (19.5%)	1 (16.7%)	23 (19.7%)	1.000
Atelectasis	12 (9.8%)	1 (16.7%)	11 (9.4%)	0.467
Others*	3 (2.4%)	1 (16.7%)	2 (1.7%)	0.140

All results are expressed as number and percentage. Some patients had more than 1 respiratory complication. AKI: acute kidney injury. *Others: upper airway edema: 1; pulmonary thromboembolism:2; water overload: 1; acute respiratory distress syndrome: 1.

**Table 4:** Outcomes.

	All (n = 123)	AKI (n = 6)	Non-AKI (n = 117)	p
Worse SOFA (median and I.R.)	2.00 (1.00-3.00)	4.50 (1.00-7.00)	2.00 (1.00-3.00)	0.056
Polyuria	105 (85.4%)	5 (83.3%)	100 (85.5%)	1.000
Vasoactive support during ICU admission	16 (13.1%)	2 (33.3%)	14 (12.1%)	0.177
Hematuria	9 (7.4%)	2 (33.3%)	7 (6.0%)	0.062
Anemia	75 (61.0%)	5 (83.3%)	70 (59.8%)	0.403
Infectious complications	10 (8.1%)	0 (0.0%)	10 (8.5%)	1.000
ICU stay, days (median and I.R.)	5.00 (4.00-5.00)	5.00 (4.50-5.00)	5.00 (3.50-5.00)	0.432
ICU mortality (n and percentage)	1 (0.8%)	0 (0.0%)	1 (0.9%)	1.000
Hospital stay, days (median and I.R.)	10.00 (8.00-13.00)	9.00 (8.25-20.00)	10.00 (8.00-13.00)	0.727
Hospital mortality (n and percentage)	1 (0.8%)	0 (0.0%)	1 (0.9%)	1.000

AKI: acute kidney injury. I. R.: Interquartile range. ICU: Intensive care unit. SOFA: Sepsis Organ Failure Assessment.

## References

[1] Dube P, Sideris L, Law C (2015). Guidelines on the use of cytoreductive surgery and hyperthermic intraperitoneal chemotherapy in patients with peritoneal surface malignancy arising from colorectal or appendiceal neoplasms. Curr Oncol.

[2] Dedrick RL, Myers CE, Bungay PM (1978). Pharmacokinetic rationale for peritoneal drug administration in the treatment of ovarian cancer. Cancer Treat Rep.

[3] Verwaal VJ, van Tinteren H, Ruth SV (2004). Toxicity of cytoreductive surgery and hyperthermic intra-peritoneal chemotherapy. J Surg Oncol.

[4] Hu J, Wang Z, Wang X (2023). Side-effects of hyperthermic intraperitoneal chemotherapy in patients with gastrointestinal cancers. PeerJ.

[5] Tan JW, Tan GHC, Ng WY (2020). High-grade complication is associated with poor overall survival after cytoreductive surgery and hyperthermic intraperitoneal chemotherapy. Int J Clin Oncol.

[6] Naffouje SA, Tulla KA, Chorley R (2018). Acute kidney injury increases the rate of major morbidities in cytoreductive surgery and HIPEC. Ann Med Surg (Lond).

[7] Charlson ME, Pompei P, Ales KL (1987). A new method of classifying prognostic comorbidity in longitudinal studies: development and validation. J Chronic Dis.

[8] Knaus WA, Draper EA, Wagner DP (1985). APACHE II: a severity of disease classification system. Crit Care Med.

[9] Vincent JL, de Mendonca A, Cantraine F (1998). Use of the SOFA score to assess the incidence of organ dysfunction/failure in intensive care units: results of a multicenter, prospective study. Working group on "sepsis-related problems" of the European Society of Intensive Care Medicine. Crit Care Med.

[10] Sugarbaker PH (1998). Intraperitoneal chemotherapy and cytoreductive surgery for the prevention and treatment of peritoneal carcinomatosis and sarcomatosis. Semin Surg Oncol.

[11] Kellum JA, Lameire N, Group KAGW (2013). Diagnosis, evaluation, and management of acute kidney injury: a KDIGO summary (Part 1). Crit Care.

[12] Cata JP, Zavala AM, Van Meter A (2018). Identification of risk factors associated with postoperative acute kidney injury after cytoreductive surgery with hyperthermic intraperitoneal chemotherapy: a retrospective study. Int J Hyperthermia.

[13] Liesenfeld LF, Wagner B, Hillebrecht HC (2022). HIPEC-Induced Acute Kidney Injury: A Retrospective Clinical Study and Preclinical Model. Ann Surg Oncol.

[14] Carias E, Ferreira H, Chuva T (2022). Acute Kidney Injury After Cytoreductive Surgery and Hyperthermic Intraperitoneal Chemotherapy in a Portuguese Population. World J Oncol.

[15] Angeles MA, Quenet F, Vieille P (2019). Predictive risk factors of acute kidney injury after cytoreductive surgery and cisplatin-based hyperthermic intra-peritoneal chemotherapy for ovarian peritoneal carcinomatosis. Int J Gynecol Cancer.

[16] Randle RW, Ahmed S, Levine EA (2015). Significance of diabetes on morbidity and mortality following cytoreductive surgery with hyperthermic intraperitoneal chemotherapy. J Surg Oncol.

[17] Sin EI, Chia CS, Tan GHC (2017). Acute kidney injury in ovarian cancer patients undergoing cytoreductive surgery and hyperthermic intra-peritoneal chemotherapy. Int J Hyperthermia.

[18] Liesenfeld LF, Quiring E, Al-Saeedi M (2023). Extensive Peritonectomy is an Independent Risk Factor for Cisplatin HIPEC-Induced Acute Kidney Injury. Ann Surg Oncol.

[19] Chen CY, Chang HY, Lu CH (2020). Risk factors of acute renal impairment after cytoreductive surgery and hyperthermic intraperitoneal chemotherapy. Int J Hyperthermia.

[20] Hakeam HA, Breakiet M, Azzam A (2014). The incidence of cisplatin nephrotoxicity post hyperthermic intraperitoneal chemotherapy (HIPEC) and cytoreductive surgery. Ren Fail.

[21] Solanki SL, Mukherjee S, Agarwal V (2019). Society of Onco-Anaesthesia and Perioperative Care consensus guidelines for perioperative management of patients for cytoreductive surgery and hyperthermic intraperitoneal chemotherapy (CRS-HIPEC). Indian J Anaesth.

[22] Bihorac A, Kellum JA (2015). Acute kidney injury in 2014: a step towards understanding mechanisms of renal repair. Nat Rev Nephrol.

[23] Liesenfeld LF, Brandl A (2023). Influence of hyperthermic intraperitoneal chemotherapy on renal blood perfusion. Langenbecks Arch Surg.

[24] Arslan NC, Sokmen S, Avkan-Oguz V (2017). Infectious Complications after Cytoreductive Surgery and Hyperthermic Intra-Peritoneal Chemotherapy. Surg Infect (Larchmt).

[25] Smibert OC, Slavin MA, Teh B (2020). Epidemiology and risks for infection following cytoreductive surgery and hyperthermic intra-peritoneal chemotherapy. Support Care Cancer.

[26] Cascales Campos P, Martinez Insfran LA, Wallace D (2020). Identifying the incidence of respiratory complications following diaphragmatic cytoreduction and hyperthermic intraoperative intraperitoneal chemotherapy. Clin Transl Oncol.

[27] Sullivan BJ, Bekhor EY, Carpiniello M (2020). Diaphragmatic Peritoneal Stripping Versus Full-Thickness Resection in CRS/HIPEC: Is There a Difference?. Ann Surg Oncol.

[28] Fan B, Bu Z, Zhang J (2021). Phase II trial of prophylactic hyperthermic intraperitoneal chemotherapy in patients with locally advanced gastric cancer after curative surgery. BMC Cancer.

[29] Yu P, Huang X, Huang L (2023). Hyperthermic intraperitoneal chemotherapy (HIPEC) plus systemic chemotherapy versus systemic chemotherapy alone in locally advanced gastric cancer after D2 radical resection: a randomized-controlled study. J Cancer Res Clin Oncol.

[30] Blum Murphy M, Ikoma N, Wang X (2020). Phase I Trial of Hyperthermic Intraperitoneal Chemoperfusion (HIPEC) with Cisplatin, Mitomycin, and Paclitaxel in Patients with Gastric Adenocarcinoma and Associated Carcinomatosis or Positive Cytology. Ann Surg Oncol.

[31] Somashekhar SP, Yethadka R, Kumar CR (2020). Toxicity profile of chemotherapy agents used in cytoreductive surgery and hyperthermic intraperitoneal chemotherapy for peritoneal surface malignancies. Eur J Surg Oncol.

[32] Schilsky RL, Anderson T (1979). Hypomagnesemia and renal magnesium wasting in patients receiving cisplatin. Ann Intern Med.

[33] Lam M, Adelstein DJ (1986). Hypomagnesemia and renal magnesium wasting in patients treated with cisplatin. Am J Kidney Dis.

[34] Cripe J, Tseng J, Eskander R (2015). Cytoreductive surgery and hyperthermic intraperitoneal chemotherapy for recurrent ovarian carcinoma: analysis of 30-day morbidity and mortality. Ann Surg Oncol.

[35] Zaballos M, Power M, Canal-Alonso MI (2021). Effect of Cilastatin-Induced Nephrotoxicity in Patients Undergoing Hyperthermic Intraperitoneal Chemotherapy. Int J Mol Sci.

